# Occurrence of an invasive coral in the southwest Atlantic and comparison with a congener suggest potential niche expansion

**DOI:** 10.1002/ece3.1506

**Published:** 2015-05-06

**Authors:** Lélis A Carlos-Júnior, Danilo M Neves, Newton P U Barbosa, Timothy P Moulton, Joel C Creed

**Affiliations:** 1 Departamento de Ecologia e Evolução, Universidade do Estado do Rio de JaneiroRua São Francisco Xavier, 524 – Maracanã, Rio de Janeiro, CEP: 20550-013, Brazil; 2 Coral-Sol Research, Technological Development and Innovation NetworkRio de Janeiro, Brazil; 3 Royal Botanic Garden Edinburgh20a Inverleith Row, Edinburgh, Midlothian, EH3 5LR, UK; 4 Departamento de Biologia Geral, Universidade Federal de Minas GeraisAvenida Antônio Carlos, 6627 – Pampulha, Belo Horizonte, 31270901, Brazil

**Keywords:** Coral species, marine invasions, niche breadth, species distribution modeling, *Tubastraea coccinea*, *Tubastraea tagusensis*

## Abstract

*Tubastraea tagusensis*, a coral native to the Galapagos Archipelago, has successfully established and invaded the Brazilian coast where it modifies native tropical rocky shore and coral reef communities. In order to understand the processes underlying the establishment of *T. tagusensis,* we tested whether Maxent, a tool for species distribution modeling, based on the native range of *T. tagusensis* correctly forecasted the invasion range of this species in Brazil. The Maxent algorithm was unable to predict the Brazilian coast as a suitable environment for the establishment of *T. tagusensis*. A comparison between these models and a principal component analysis (PCA) allowed us to examine the environmental dissimilarity between the two occupied regions (native and invaded) and to assess the species' occupied niche breadth. According to the PCA results, lower levels of chlorophyll-*a* and nitrate on the Atlantic coast segregate the Brazilian and Galapagos environments, implying that *T. tagusensis* may have expanded its realized niche during the invasion process. We tested the possible realized niche expansion in *T. tagusensis* by assuming that *Tubastraea* spp. have similar fundamental niches, which was supported by exploring the environmental space of *T. coccinea,* a tropical-cosmopolitan congener of *T. tagusensis*. We believe that the usage of Maxent should be treated with caution, especially when applied to biological invasion (or climate change) scenarios where the target species has a highly localized native (original) distribution, which may represent only a small portion of its fundamental niche, and therefore a violation of a SDM assumption.

## Introduction

Biological invasions are one of the biggest conservation concerns and have profound impacts in an integrated global society (Aguin-Pombo et al. 2012). In marine environments, invasive species threaten biodiversity, the economy (including fisheries and tourism), and human health (Bax et al. 2003; Sorte et al. 2010). Much effort by the scientific community has been focused on providing information that can be used to prevent such invasion events or manage them. In this context, innovative computational tools capable of predicting species distributions soon became popular in studies of biological invasions (Jiménez-Valverde et al. 2011).

These tools, often called species distribution models (hereafter SDMs), which include Maxent (see below), yield potential distributional maps of a given species based on the environmental conditions (or climatic envelopes) associated with the species presence (Corsi et al. 2000; Peterson and Shaw 2003). Considering the species environmental conditions requirements as part of its niche (Grinnell 1917; Hutchinson 1957), the use of SDMs for predicting invasion assumes that the species maintains its niche across space during the process of invasion (Broennimann 2007; Pearman et al. 2008; Peterson 2011).

If this niche persistence assumption is violated, that is, if a change occurs in the species' observed niche during the invasion process, the use of Maxent for predicting invasions may be compromised (Rödder and Lötters 2009). This is especially critical in cases where only the native occurrence range of the species is well known (for example, when considering risk assessment of invasion potential into new regions), or, more likely, when the invasion has just begun and data on the invasion distribution range are limited (Broennimann and Guisan 2008; Anderson and Raza 2010). Moreover, observed niche variation has been suggested to occur in invasion events (Broennimann et al. 2007; Rödder and Lötters 2009; but see Guisan et al. 2014) and thus understanding whether a given species maintains its niche breadth or not is crucial to assess the usefulness of a particular SDM in predicting invasions and thus for conservation.

**Figure 1:**
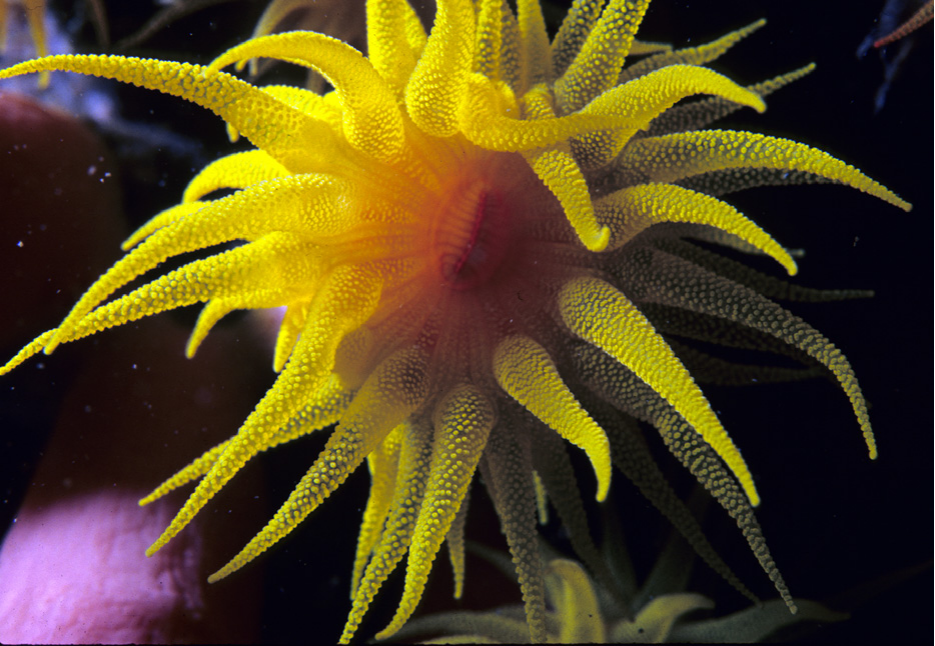
The invasive cup coral *Tubastraea tagusensis*.

The scleractinian *Tubastraea tagusensis* (Fig.1) is an azooxanthellate and ahermatypic coral species endemic to the Galapagos archipelago. Even in the archipelago this species is restricted to shallow waters along the coasts of certain islands (Wells 1982), but in the early 2000s, *T. tagusensis* was reported on the South Atlantic coastline of Brazil as a nonindigenous species (de Paula and Creed 2004) and soon expanded its range. Today its range reaches over 2000 km along the Brazilian coast. *T. tagusensis* is capable of outcompeting local organisms, including endemic species (Creed 2006). Another species from the Pacific, *T. coccinea*, has also invaded the Atlantic reaching Brazil, the Gulf of Mexico, and the Caribbean Sea, with occurrences in Texas and Florida (USA) (Fenner and Banks 2004; de Paula and Creed 2004; Sammarco et al. 2004). Unlike its congener, *T. coccinea* is more broadly distributed through its native Indo-Pacific region (Cairns 2000).

Our goal was to assess what are the main environmental factors driving the successful invasion of the originally narrowly distributed species *T. tagusensis* throughout the Brazilian coast. We also investigated whether it would be possible to predict the invasion of *T. tagusensis* in Brazil using only its native distribution as the predictor to feed the model, as information on the invaded range of a newly introduced species is usually limited and so it is commonplace for models to make predictions using only the available native occurrence records. As the native distribution of *T. tagusensis* is quite narrow, we also tested whether model predictions for the broadly distributed and also invasive congener *T. coccinea* were capable of predicting both species' invasion, using it as a proxy for the genus, in order to better understand the distributional aspects and species specificities of the invasion of the genus *Tubastraea* into the Atlantic.

## Materials and Methods

### Selection of a species distribution model

Many SDMs consist of algorithms capable of providing a potential distribution map of a given species, associating its occurrence (geographical coordinate) data with environmental conditions extracted from those occurrence points (Anderson and Raza 2010). The assessment of biological variable values associated with the presence of the species provides the potential suitability of a given location to the species occurrence (Peterson 2003). As our correlative modeling algorithm, we chose Maxent 3.3.3a, because this presence-background tool (Phillips et al. 2006; Phillips and Dudik 2008) has been shown to perform well in comparative studies (Elith et al. 2006; Hernandez et al. 2006; Wisz et al. 2008; ). Furthermore, this method has also performed well in previous studies of marine species, like stony coral species (Tittensor et al. 2009), and outperforms other algorithms when modeling with few species records with restricted distributions, as is the case for *T. tagusensis* (Hernandez et al. 2006). The basic output of Maxent is intuitive probabilities of occurrence estimated from a set of environmental layers (Phillips and Dudik 2008). Maxent estimates a species' environmental niche by finding the distribution closest to uniform when the expected value for each value (*i.e*., environmental variable) under the estimated distribution matches its empirical average. This approach is called maximum entropy and it basically finds a maximum-likelihood distribution for the species considering the given environmental information at the presence points of the species, given as geographic coordinates (Phillips et al. 2004).

### Species occurrence data

We combined the records containing the species occurrence as geographical coordinates available in the literature with online databases (Ocean Biogeographic Information System (OBIS – http://www.iobis.org/ last accessed in November 2014) (Vanden Berghe 2007), Global Biodiversity Information Facility (GBIF – http://www.gbif.org, last accessed in November 2014), and the Cria species Link (http://www.splink.org.br, last accessed in May 2012)) to find 11 points of occurrence for *Tubastraea tagusensis* in the Galapagos Archipelago, which is a small but sufficient number of records to model in Maxent (Hernandez et al. 2006; Pearson et al. 2007). We used the same abovementioned online data sources to obtain 57 occurrence records for the cosmopolitan sibling species *Tubastraea coccinea*, Lesson 1829. These data were used to compare the occupied environmental range of the congeners.

### Environmental variables

We extracted the environmental variables from available on Bio-Oracle marine dataset (Tyberghein et al. 2011). It comprises 23 variables in GIS-based raster grids with a 5 arcmin (approximately 9.2 km) spatial resolution and performs well in explaining the distribution of marine organisms (Tyberghein et al. 2011). These raster files were managed in Arc-Gis 9.3 to provide masks for the targeted regions of the globe. To avoid overparameterized analyses (Ginzburg and Jensen 2004), we selected a subset of predicting variables based on a correlation level threshold (*r* = 0.85) and on exploratory analyses. This cutoff was chosen following intermediate and similar procedures described in other studies (Rissler and Apodaca 2007; Werneck et al. 2011) in which even variables with *r* > 0.50 should not be excluded a priori (Drake et al. 2006). The selected variables were mean calcite concentration (calcite, mol/m³), maximum photosynthetically available radiation (parmax, Einstein/m²/day), mean pH (pH), mean salinity (salinity, PPS), mean nitrate concentration (nitrate, *μ*mol/L), and maximum chlorophyll-*a* concentration (chlomax, mg/m³). Despite their general importance to the distribution of marine organisms, mean, maximum, range, and minimum temperatures were among the excluded variables due to their poor individual contribution to model gain in preliminary training models.

Although the six selected variables were selected to explore the environmental occupied niche of the two species (see “Principal Component Analysis” section below), the relatively small number of *T. tagusensis* occurrence records (*n *=* *11) limits the use of them in the Maxent model. The excess of predictors on a SDM leads to *overfitting* (Warren and Seifert 2011), a methodological bias that undermines confidence on the transferability of the model, particularly in studies when the goal is to project the distribution of a species from one place to another (as in our case) (Beaumont et al. 2005; Peterson et al. 2007; Radosavljevic and Anderson 2014). In fact, our first exploratory models using all the variables and different regularization multipliers (indicated as a good way to search for *overfitting*; see Warren and Seifert 2011 and Radosavljevic and Anderson 2014 for details) suggested *overfitting* on the models that used more parameters. This reinforces the importance of variable selection for modeling assessments. Thus, two variables were chosen based on the importance of each variable to model gain in those aforementioned exploratory models and the knowledge of the authors regarding the biology of the species. The first was chlomax, which serves a proxy for community type, because it measures the quantity of phytoplankton on the water. The second was mean nitrate concentration, as a limiting nutrient for marine organisms.

### SDM evaluation

For the native areas where the Maxent algorithm was calibrated, 75% of the occurrence records were used for model development and the remaining 25% of the data set was used to evaluate model performance. For projected areas (*i.e*., the invaded regions), we used the entire set of native region occurrences to develop the model and the known records from the Brazilian coast for model evaluation. In both scenarios, we used the area under curve (AUC test) for model evaluation. AUC test comprises a threshold-independent measure of model performance as compared with the null hypothesis for the prediction (Fielding and Bell 1997). When the AUC is ≤ 0.50, the model performance is considered to be low, no better than random prediction, and higher AUC values indicate better prediction results. We used minimum training presence as our convergence threshold and performed 11 bootstrapped replicates.

### Principal component analysis

Principal components analysis (PCA) was the ordination method applied in this study. This distance-based metric was generated using the R statistical program with the analytical package stats (R Development Core Team, 2014). For the PCA, we gathered occurrence data of *T. tagusensis* for both native and invaded regions. Since 2000, the *Consorcio Projeto Coral-Sol* (Sun-Coral Project Consortium – Instituto Brasileiro de Biodiversidade and Fundação OndAzul) has been monitoring *Tubastraea* spp. and maintains the National Sun-Coral Database from which occurrence data were extracted. Our final PCA matrix consisted of 29 *T. tagusensis* records for Brazil, 11 points from Galapagos and the six selected environmental variables. Furthermore, we tested the possible realized niche expansion in *T. tagusensis* (Peterson 2011) by assuming that *Tubastraea* spp. have similar fundamental niches, which is common between sibling species. Thus, in order to explore this assumption, we used 57 occurrence records of *T. coccinea,* the tropical-cosmopolitan congener of *T. tagusensis*, from its native region, the Indo-Pacific. These were obtained from the online databases cited above (see section: Species occurrence data).

We also used the framework protocol suggested by Guisan et al. (2014) in order to further explore the niche variations shown in the PCA. This framework is useful to decompose the various elements of a niche change and to objectively calculate niche expansion. The so-called COUE scheme (from Centroid, Overlap, Unfilling and Expansion) allowed us to determine the change in mean niche position by Centroid (C) measures, nonindigenous niche Expansion (E) or Unfilling (U) when compared to the native range and, finally, niche stability (S*p*) of pooled range spaces between the two ranges. For our purposes, S*p* is equal to the Overlap (O) between those two ranges and measures the amount of superposition between two distributions. The overlapping ratio is given by the proportion of the entire pool of occurrences of the species present in both ranges, native and nonindigenous, which may be considered as a surrogate for niche maintenance, or stability, during the invasion. Centroid shifts indicate change in mean niche position and Unfilling or Expansion can be considered to be the nonoverlapping parts of two niches and are informative measures when considering the relative change between the nonindigenous and the native ranges of a given species. Thus, while S*p* or O is measures of stability, U and E are a proxy for detecting the extent of change between two distributions. For a full description of the methods and terminology, see Guisan et al. (2014).

## Results

**Figure 2:**
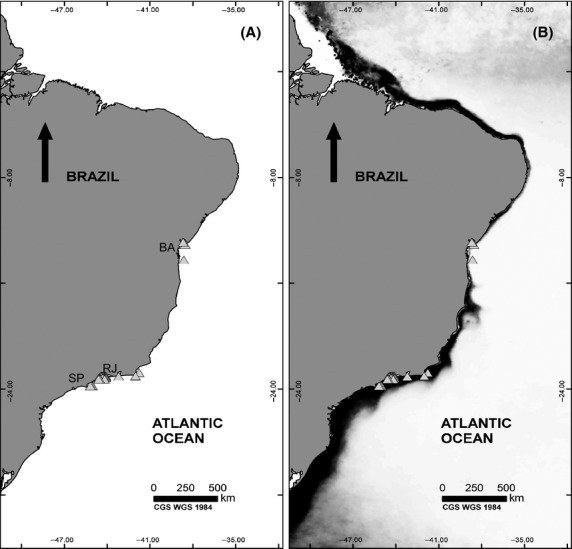
Distribution maps for the invasive corals *Tubastraea* spp. (A) Potential distribution map of *T. tagusensis* for the Brazilian coast using only native points to feed the model. White area represents environmental suitability below 10%. This predicted low conformity between the area conditions and the species niche is contrasted with the actual occurrence and settlement of several populations of *T. tagusensis* in Brazil (gray triangles) and (B) native occurrence records of *T. coccinea* used to predict its own potential distribution in Brazil. Coastal areas with environmental suitability above 80% are shown in black, and areas with suitability above 70% are shown in dark gray. *T. tagusensis* presence records are shown as gray triangles.

It was possible to develop highly predictive models for the Galapagos Archipelago using 75% of the native occurrence records to predict the presence of *T. tagusensis* in the area (AUC = 0.96). Nevertheless, using only native occurrence record data, the potential distribution model of *T. tagusensis* predicts no environmental suitability for the species on the southern Atlantic coast of Brazil (Fig.2A).

**Figure 3:**
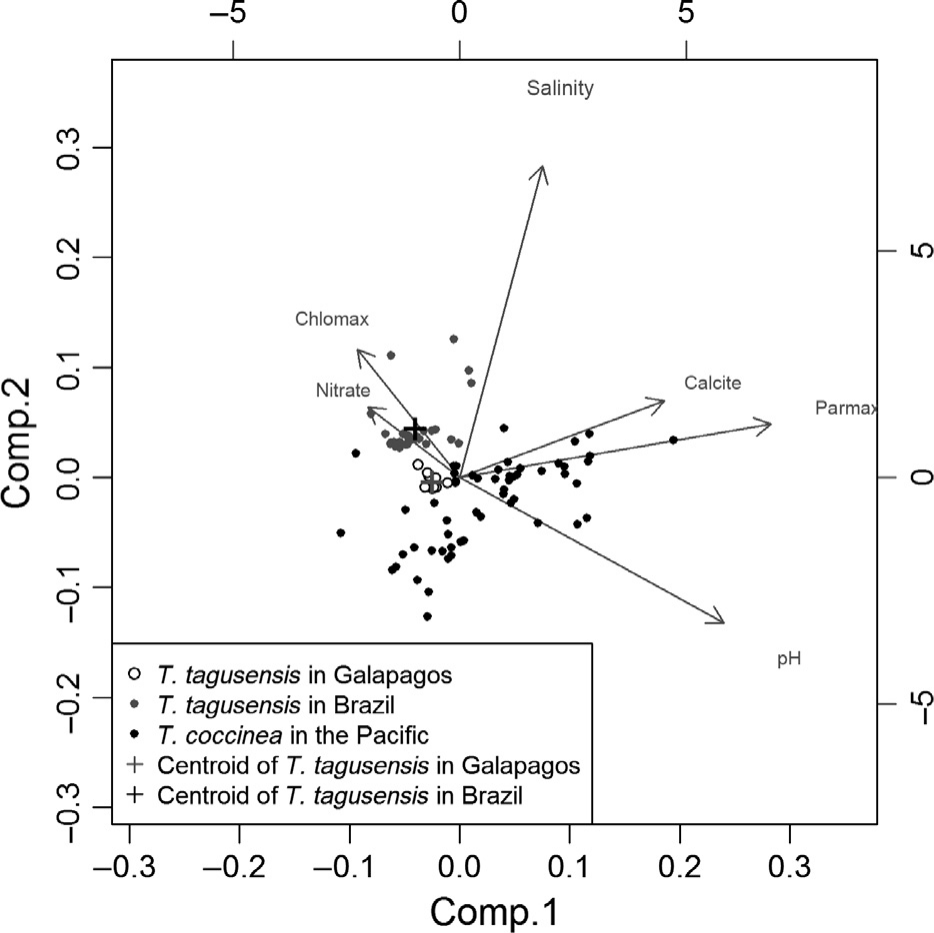
Principal components analysis of abiotic variables from the occurrence records of the *Tubastraea coccinea* (Indo-Pacific Ocean) and *T. tagusensis* (Brazil and Galapagos Archipelago) populations.

The first two PCA axes explained 33% of the variation in the environmental data. Axis 2 of the PCA was effective in segregating the Brazilian and Galapagos environments, which partially explains the modeled prediction failure (Fig.3). Maximum chlorophyll, mean nitrate and mean salinity gradients explained most of the variation. Overall, for the second axis, there was no overlap between the two environments (Galapagos records *vs*. Brazilian records). Therefore, E = 1 and U = 1; while S*p *= 0. On the other hand, the 57 Indo-Pacific occurrence records of *T. coccinea* are broadly spread along both axes and some of the points overlap both the Galapagos and the Brazilian ranges. The variables responsible for segregating the native and invaded ranges of the species are the same variables (chlomax and nitrate) selected to model the species. In addition, the model using the native occurrence records of *T. coccinea* not only successfully predicts the species invasion in Brazil (AUC test= 0.95) but is also capable of predicting the occurrence of its congener (*T. tagusensis*) in Brazil (AUC test = 0.99; Fig.2B). This is consistent with our field observations in Brazil, where we find that the two species usually coexist when present at the same sites.

## Discussion

Our analyses show that based on the abiotic conditions from the native region of *T. tagusensis*, the potential distribution model does not predict environmental suitability for this coral on the southern Atlantic coast. Wide tolerance to environmental conditions is a common feature of successful invaders (Miller et al. 2007; Küster et al. 2008), and *Tubastraea* has shown a wide tolerance to temperature, occurring in both tropical warm waters and temperate regions or even in upwelling colder water regions (Cairns 2000; Paula and Creed 2005; Paz-García et al. 2007; Glynn et al. 2008). This could explain the relative unimportance of temperature to the species modeling.

*Tubastraea tagusensis* was first recorded in Rio de Janeiro on tropical rocky shores (RJ Fig.2A) where it has successfully invaded and occupied the coast (Castro and Pires 2001; de Paula and Creed 2004; Mizrahi 2008) and where monitoring has shown that the populations are well established (Silva et al. 2014). In Rio de Janeiro State, these shores undergo sporadic localized coastal upwelling. Other records have been reported further south on subtropical rocky shores (São Paulo state – SP) (Mantelatto et al. 2011) and north on coral reefs (Bahia state – BA) (Fig.2) (Sampaio et al. 2012) so the range occupied by *T. tagusensis* in the invaded regions is quite substantial.

We identify three explanations for such a prediction failure: (1) the package of abiotic variables used is not suitable for our modeling objectives, (2) the environmental layers used to generate the models could be incapable of explaining the abiotic requirements of *T. tagusensis* (the distribution of this species could be either regulated by environmental conditions different from those used in our modeling approach, biotic interactions, or by stochasticity), and (3) the broad environmental requirement of the species allows it to be successfully established in two environmentally distinct regions (the Galapagos Pacific region and the southern Atlantic coast of Brazil).

We consider the first explanation unlikely as: (1) exploratory models fed with different sets of variables yielded similar results, (2) the data source used to generate both models has been broadly and successfully used to yield distribution maps of several marine organisms (Tyberghein et al. 2011) including corals, and (3) the model generated for *T. coccinea*, the tropical-cosmopolitan congener of *T. tagusensis*, was successful in predicting both its own occurrence and also the occurrence of *T. tagusensis* in the southwest Atlantic (Fig.2B). It is highly unlikely that the environmental layers are irrelevant for *T. tagusensis*. Moreover, the training model (using 75% of occurrences to train the model and 25% to test it) successfully predicted the native distributional range of *T. tagusensis* in Galapagos (AUC = 0.96) indicating that it is unlikely that the variables used were not relevant for the species.

Due to its oceanographic settling, the marine environment in Galapagos is unique and variable. This is due to the equatorial upwelling of cool, nutrient-rich water which affects the entire archipelago (Houvenaghel 1978; Wyrtki 1981) being punctuated by highly irregular (scale of several years) effects of *El Niño* Southern Oscillation (ENSO) events which may cease equatorial upwelling and cause sudden extreme changes in surface waters. These changes impact the archipelago's marine community, including corals, which is subjected to wide fluctuation in many abiotic variables (Glynn and de Weerdt 1991; Witman and Smith 2003). Although the Bio-Oracle marine dataset contains some range variables (Tybergh-ein et al. 2011), our model might not have adequately captured the temporal variability inherent in the oceanographic setting in which the species occurs. This mismatch could explain why the model did not predict the successful invasion of *T. tagusensis* in Brazil.

Despite the high seasonal variability intrinsic to the archipelago's oceanography, the failure of the model in predicting the known habitat suitability in Brazil might also be explained by real spatial environmental dissimilarities between the native and invaded ranges of the species. Thus, colonizing and establishing in Brazil represented a spatial expansion of the observed niche of *T. tagusensis*. It is important to note that the second and third explanations are not mutually exclusive as the irregular annual-decadal instability of environmental conditions in the Galapagos Archipelago may have selected euryoecious organisms capable of inhabiting and invading different environments.

Interestingly, in its native range in the Galapagos Archipelago, *T. tagusensis* is restricted to certain islands (Wells 1982). Theoretically, restricted endemic organisms are expected to have very specific habitat requirements, a fact taken into account, for example, in predicting extinctions in climate changing scenarios (Thomas et al. 2004; Malcolm et al. 2006). Indeed after a particularly severe ENSO event in 1982–1983, *T. tagusensis* was thought to have become extinct in the Galapagos (Glynn and de Weerdt 1991), but re-established subsequently. Nevertheless, narrow distribution ranges are not necessarily associated with strict climatic requirements, as seems to be the case for *T. tagusensis*. Some originally restricted species can present broader niche breadths, as already observed, for example, in trees and birds (Schwartz et al. 2006) and frogs (Williams et al. 2006). In the former study, 87% of the endangered plant species, all endemic to Florida, may have been poorly designated as threatened by assuming that their current restricted range reflects narrow environmental tolerances. The highly localized native distribution of *T. tagusensis* is intriguing and may reflect the interaction of sporadic climatic effects, limitation of dispersion, and/or limitations of biotic interactions (e.g., competition or predation) (see Edgar et al. 2010) rather than restrictive nonsuitable environmental conditions.

In Brazil, human transportation vectors have helped the species to overcome the dispersal barriers that might constrain it in its native environment. Moreover, the receptor community lacks natural predators of *Tubastraea* (Moreira and Creed 2012). Thus, although *T. tagusensis* might be restricted in the Galapagos by localized biotic or dispersal limitation constraints, in Brazil it may expand its geographical range unchecked. The PCA showed dissimilarity between the Galapagos and the Brazilian environments for the occurrence of *T. tagusensis*. Seeing as *T. tagusensis* has successfully invaded the Brazilian coast, this ordination result supports a wider environmental range of *T. tagusensis*, because the native “climatic envelope” occupied by this species is clearly distinct from the invaded environment. According to the studies of Broennimann et al. (2007), Rödder and Lötters (2009), and Medley (2010) this mismatch is indicative of a species with a broad *fundamental* niche breadth, but it is also clear evidence of a *realized* niche expansion during the process of invasion and establishment into a new region. Sometimes the climate envelope in the nonindigenous range poorly represents the native environment (Soberon and Townsend Peterson 2011; Guisan et al. 2014). When this is modeled and projected, the consequent displacement of the species distributional cloud onto the nonindigenous range could lead to a false impression of evolutionary “niche shift”.

The niche expansion of *T. tagusensis* reflects the enlargement in the realized niche of the species (Broennimann et al. 2007). Unlike *T. tagusensis*, *T. coccinea* is a cosmopolitan species occurring throughout the Pacific (Cairns 1994). Its wide native range and corresponding environmental conditions have allowed it to successfully invade the tropical southwest Atlantic and Caribbean Sea (Cairns 2000; Fenner 2001; Fenner and Banks 2004). If these *Tubastraea* spp. have similar fundamental niches, a common trait between sibling species (see the Niche Conservatism Hypothesis, Peterson 2011), the successful invasion of *T. tagusensis* could be due to an expansion in its realized niche. If *T. tagusensis* has a broad *fundamental* niche breadth shared with its congener *T. coccinea*, this would allow the observed *realized* niche to vary during the process of invasion in Brazil. This is consistent with the PCA results. In Brazil, the two species frequently co-occur, sometimes physically fusing their colonies, and although a number of comparative studies have been carried out, only small differences in traits have been identified, such as in substratum preference and sexual maturation periods (Mangelli and Creed 2012; de Paula et al. 2014).

The genus *Tubastraea* is generally rare in areas with dense and diverse coral populations in the Pacific (Wood 1983), whereas in Brazil, *T. tagusensis* can become dominant, outcompetes native corals (Creed 2006), and has no effective predators (Moreira and Creed 2012). This enemy release (Crawley 1987; Keane and Crawley 2002) is another determinant of the successful expansion of *T. tagusensis*. The co-occurrence of ecological and evolutionary processes seems to be the most parsimonious explanation for the niche shift observed and the invasive success of *T. tagusensis* (Dietz and Edwards 2006; Van Kleunen et al. 2010).

This niche expansion highlights the need for caution in using modeling techniques such as Maxent in climate change scenarios (*e.g.,* Jueterbock et al. 2013), where potentially false assumptions of steadiness of the environmental requirements of the species (in space and time) may result in erroneous predictions and misinterpretation of potential impacts (Schwartz et al. 2006; Rödder and Lötters 2009). This study suggests that predicting species invasion using “climatic envelopes” in Maxent can be particularly tricky or even misleading when dealing with species with limited native distributions and few records from the non-native range or when only the native range occurrence data are available (Fitzpatrick et al. 2007; Broennimann and Guisan 2008; Jiménez-Valverde et al. 2011). In predictive studies of biological invasions, such problems can lead to poor risk assessments and potentially ineffective conservation strategies, resulting in economical and ecological damage (Lockwood et al. 2007).
